# The involvement of older inpatients in medical student education

**DOI:** 10.1007/s41999-017-0023-1

**Published:** 2018-01-24

**Authors:** T. Aquilina, S. M. Thompson, K. H. M. Metcalfe, H. Hughes, L. Sinclair, F. Batt

**Affiliations:** 1 Oxford University Medical School, Oxford, UK; 2 Departments of General (Internal) Medicine and Geratology, John Radcliffe Hospital, Oxford, UK

**Keywords:** Older inpatients experience, Medical students education, Patient/student interaction

## Abstract

**Objectives:**

To examine older inpatients’ experiences with medical student education, their views on future interactions, and to seek their opinion on the most important curricular topics related to geriatric medicine.

**Methods:**

The study involved 112 non-confused inpatients older than 65 years of age, who completed a uniformed questionnaire on the day of their discharge from a teaching hospital.

**Results:**

The mean age was 81 years, with equal number of male and female participants. 57% interacted with the students during their admission, the majority being interviewed and examined. Almost all (92%) of these patients described their experience as positive, some described it as time-consuming (23%), repetitive (19%) and tiresome (9%). 92% of all participants agreed that the older patients should be part of medical students’ education. Dementia, cardiac conditions, cancer, arthritis, isolation/loneliness were highlighted as the most important topics to teach medical students related to geriatric medicine, while patience and listening were listed as important skills. They suggested practical, easily implemented advice for the improvement of the interaction between students and older patients; including allowing more time for interactions and for students to speak louder.

**Conclusions:**

Older patients felt positively about their interactions with medical students, and believed that older patients should be involved in medical student education. As well as medical conditions such as dementia, cardiac disease and cancer, these patients highlighted isolation and loneliness as important topics for undergraduate geriatric medical education, implying that students should learn about broader aspects of older patients’ health and wellbeing.

## Introduction

The trend of increasing patients’ involvement in their own care and in medical student education continues [1, 2]. It aims to improve patients’ quality of care, as well as student experience and professional development [3, 4].

University Hospitals attribute their attractiveness to patients to their staff’s greater knowledge, acquired through their teaching roles [5], while Medical Schools foster patient/student interaction. Patients, despite being central to medical education, at times provide passive illustrations of interesting conditions or contribute to the students’ experiential learning when opportunistically available in hospitals [6, 7]. During undergraduate medical education patient interactions, partly due to their authenticity, were shown to benefit students and are judged by some to be indispensable [8].

However, the learning on actual patients has also been described as “inappropriate” for the twenty-first century, and ethical questions about the use of patients as a training resource have been raised [9, 10]. Simulation-based education can help with these issues and protect patients from any possible risks [11], but rationale for the active involvement of patients in health professional education remains, and include benefits to patients and students [1, 12, 13].

At present older patients make up an increasing proportion of hospital admissions, and also participate more in undergraduate education, however little is known about their attitude and experiences related to such educational activities. We conducted a survey on older patients’ involvement and experience in medical student education during their hospital stay at the The Oxford University Hospitals Trust, John Radcliffe Hospital (OUHT, JRH). It explored their views on future involvement in medical student education, as well as their opinion on which skills and topics they believed are of particular importance for undergraduate medical education related to geriatric medicine.

## Methods

During the 4th, 5th and 6th years of their Oxford medical degree, students spend the majority of their time at OUHT, which provides services for a population of approximately 500,000, with an unselected medical and surgical admission system. The size of the population of inpatients over the age of 65 during the time of our survey in OUHT, JR was 1290 (13 wards). Students are in direct contact with patients who consent to the students’ involvement. They participate in patient care, bedside teaching, taking patient histories, as well as performing examinations and procedures such as venepuncture and peripheral venous cannulation, under supervision. The patients for the study were recruited on their discharge day at the randomly selected wards (5 wards), where the students attend their teaching; at the acute general and geriatric medical wards, surgical wards, and at the hospital discharge lounge.

The study was conducted between 03/10/2015 and 19/03/2016 on random days depending on the investigators’ availability to administer the survey. The patients eligible for the study were older than 65 years, not confused, as confirmed with the Abbreviated Mental Test Score (AMTS) of > 8, (a brief test of cognitive function in the general hospital which defines cognitive impairment if AMTS < 9), and capable of communicating their verbal consent. As older patients admitted to the OUHT, JRH have cognitive screening on admission, the notes of all older wards inpatients were screened for the AMTS by the investigators and every eligible patient was approached to participate in the study. They were asked to complete a survey that contained various questions relating to their experience with medical students during their admission, as well as their thoughts on potential interactions in the future. There was a mix of multiple choice questions, rating scale questions and open-ended questions. Only a small number refused to participate in the study, the reasons were not investigated. 112 patients completed the questionnaire after giving verbal consent. The study was granted audit approval, and quantitative data was analysed using the SPSS software package. Qualitative data was coded into themes for analysis.

## Results

The mean age of patients was 81 years; 49% male, 49% female, 2% did not state. The majority of patients (90%) were white British, slightly higher compared to the UK 2011 Census data (87%). 4.5% did not state their ethnicity, and several patients gave answers about their nationality, listing that they are Scottish, Welsh, South African, Afro-Caribbean and Cypriot. The most common reasons for admission were falls, collapse, infections, shortness of breath and gastro-intestinal (GI) related problems (non-specific abdominal pain and problems related to hernia, gallstones, GI bleeds and bowel obstruction). Their health self-rating status included 1–5 (1 very poor, 5 very good) and ranged from 1 (3%), 2 (8%), 3 (43%), 4 (30%), 5 (16%). The mean length of hospital stay was 6.1 days.

**Fig. 1:**
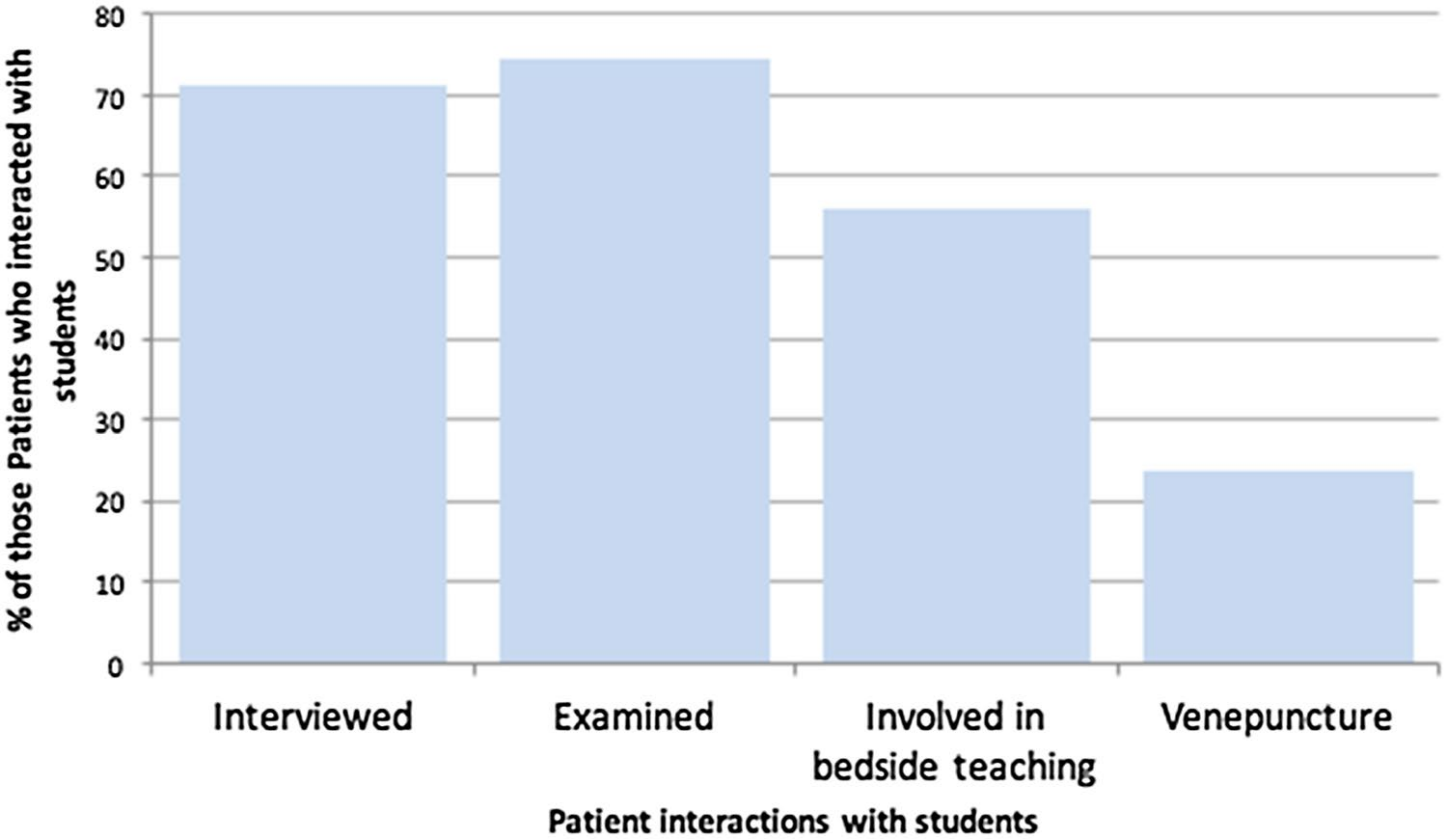
The most common interactions older patients had with medical students during this admission

59 (57%) patients were aware of medical students being involved in some way with them during their admission, and were most commonly interviewed and examined by the students, while an invasive procedure, venepuncture, was less commonly performed (Fig. 1). 9 (8%) patients were not sure if they interacted with a medical student, and 39 (35%) did not interact with a medical student.

82% of patients who interacted with students during their admission would agree to have their blood taken by a student in the future, while in those patients who did not interact with students only 61% would agree (*p* < 0.02). For those who would not agree, explanations include: “shallow veins”, “I am worried the blood may not be taken properly”, “I am scared of needles”, “They are not experienced and I do not want any pain caused by blood tests”. Patients who interacted with students were also more likely to agree to being examined (94 vs 77% *p* < 0.02) and interviewed (97 vs 80% *p* < 0.01) in the future, but not more likely to agree to be involved in bedside teaching (92 vs 80% *p* > 0.05).

The majority of all patients (92%) either ‘agreed’ or ‘strongly agreed’ with the statement ‘Older patients should be part of medical students training’. A variety of reasons were given, 28% of patients saying that “students had to learn” or similar, while some (9%) made reference to the ageing population. Other common reasons given include: “older patients are more experienced”, “older people often have lots of health problems” and “to broaden training and understanding”. 3.5% of patients disagreed or strongly disagreed and did not want to interact with students in the future in any way. Reasons included: “I don’t want students involved, I would prefer to see a doctor if I am ill” and another said “Students can learn more from other patients”. 3.5% of patients were ‘unsure’ whether they agreed or disagreed, and 1% left this question blank.

**Fig. 2:**
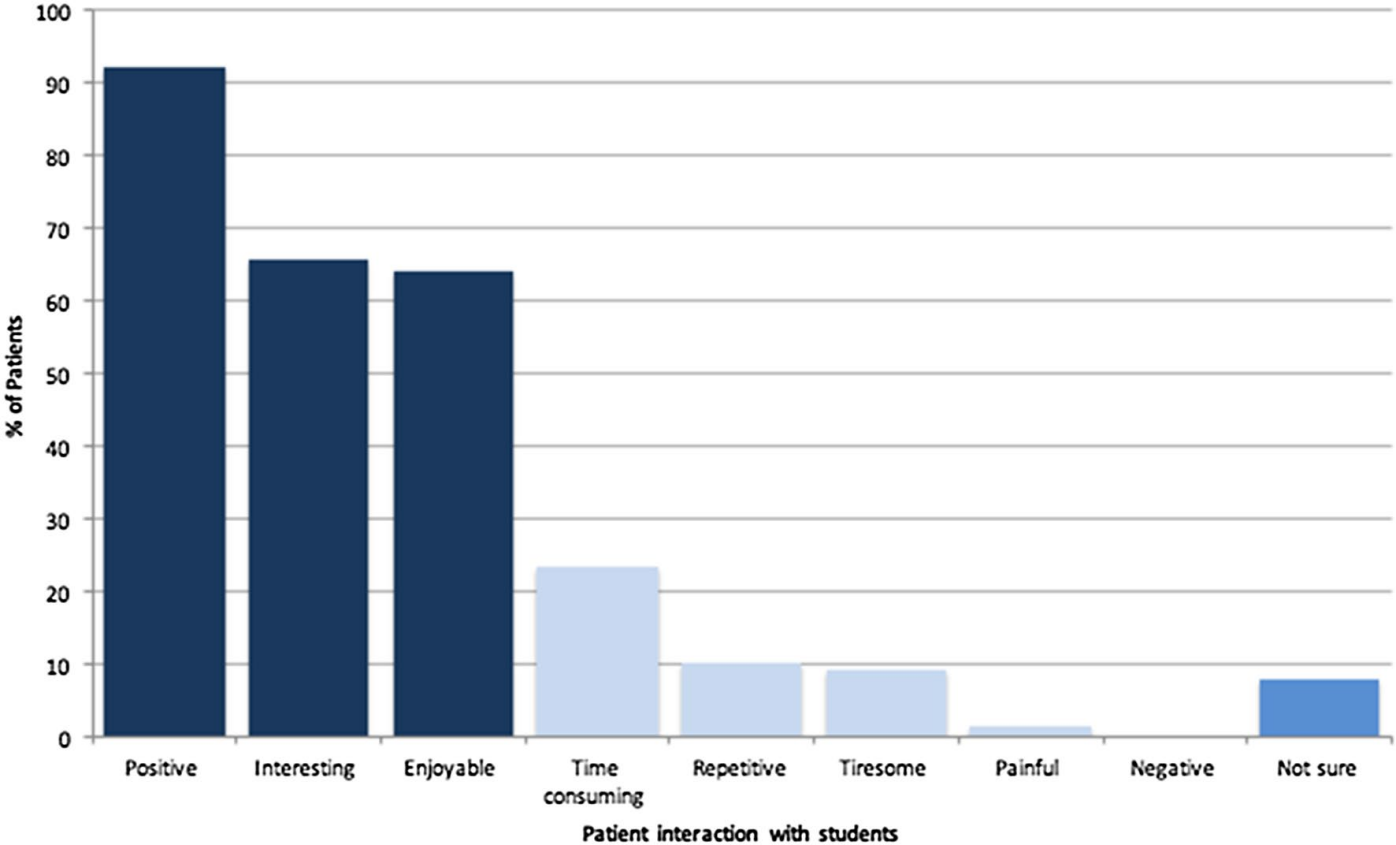
How patients describe their experience with students

Of the patients who interacted with medical students in some way during their admission, 93% felt useful to the students’ training and 92% described their experience as ‘positive’ (Fig. 2). Less favourable comments regarding their interactions included: ‘time consuming’ 23%, ‘repetitive’ 19%, and ‘tiresome’ 9%. No patients described their experience as a ‘negative’, despite one patient describing it as ‘painful’ while a blood sample was taken by a student—the same patient describes other multiple interactions (examination, interviews, bedside teaching) and was still happy to participate in bedside teaching and have blood taken by a student in future. Patients who described their experience with students as ‘interesting’ were more likely to agree to be examined in the future (Chi squared test *p* < 0.05).

The most frequent topics that patients thought were important for undergraduate students to learn about older patients are listed in Table 1. Dementia, Heart conditions and Cancer were the most commonly chosen topics. 29% of patients mentioned at least one of their own conditions. Loneliness and isolation were also listed as topics to teach medical students. Although not specifically asked about important skills for medical students, 8 (7%) patients mentioned ‘listening’ and 6 (5%) mentioned ‘patience’ as important. When asked how their interactions with medical students could have been improved, answers included: “Allow more time for students to examine”, “Be more assertive”, “Explain things better”, “Speak louder”, and “Try to listen and understand patients”.

**Table 1:** Conditions that older patients suggested it would be important for students to learn during geriatrics (some patients suggested more than one condition)

Condition	Number of patients	%
Dementia	33	29
Heart conditions	23	21
Cancer	18	16
Arthritis	14	13
Respiratory conditions	11	10
Diabetes	8	7
Blood pressure	6	5
Ageing	5	4
Falls	4	4
Isolation/loneliness	3	3

## Discussion

Only 57% of patients were sure of medical students being involved in some way with them during their admission. Students usually interact with the patients who display educational physical signs or medical history, so not every inpatient is asked to participate in student teaching. There are some days with no students on the wards due to other teaching commitments. It is possible that some patients did not realize they were interacting with students, as they dress and behave in a similar way to the doctors, and are of a similar age, although they do wear orange badges stating they are students and are introduced to the patients as students.

Our cohort of patients were younger, with slightly more male participants when compared to other hospital surveys (84 years, 44% male patients), which could influence our results, as more negative patients attitude towards involving medical students in their care was observed in younger, female patients [14, 15].

The data on the ethnicity was not complete, however higher proportion of White British inpatient population may have influenced the results, knowing that the White-British population appears to be more positive towards medical student participation, when compared to the non-White-British population [16].

It was not surprising that only the minority of patients, 11%, rated their health very poor/poor, as the interviewed patients were well enough to be discharged on that day. This may have the implications on their positive attitude to their participation in the medical students education.

Most patients described their experience as positive, none described their experience as negative. Patients who did spend time with students were more willing to be examined, interviewed or have blood taken in the future than those who did not. This suggests that patients who feel that they had gained something from their interaction with the students are more receptive to further interactions. Patients who seemed to have had less positive experience with students were not less likely to agree to see students in the future. This indicates patients accept that students need to learn and are understanding that students may not get everything right.

The advice on the improvement in interaction between students and older patients was encouraging, practical, and easily implemented; suggesting longer time for the interaction, and for students to be more assertive and speak louder.

Almost all participants thought older patients should be a part of medical students’ training, with many stating that “students had to learn”, implying that students interacting directly with older patients is necessary for the training process. Some commented on the ageing population implying that it is more important than ever for students to learn from the older patients. They also said that older patients are more experienced, who often have multiple health problems, implying that students needed to interact with older patients in order to learn about their more complex health needs. They were more willing to be involved with interviews or to be examined than to have blood taken, with some having concerns that a student taking bloods might not do it properly. This is understandable; an interview or examination is non-invasive whereas venepuncture has the possibility of being painful and repeated attempts may be needed. They seemed very aware of this—but also saying that a student taking blood would be acceptable if supervised. This shows that while older patients are willing to help students learn, they may be less willing if they feel their treatment is being affected or that their experience might cause discomfort. These results are similar to the other studies, where minority of patients describe their future participation conditional and raise potential concerns, for example about confidentiality, the nature of presenting complaints, and consent obtaining [17, 18].

When asked about conditions in geriatric medicine that students should learn about, patients listed most frequently: dementia, heart conditions and cancer. This is not an unexpected list, as these conditions cause a large burden of disease in the population of older people, however, surprisingly only a minority mentioned falls and ageing. They also identified isolation and loneliness, suggesting that patients believe medical students need to learn about broader aspects of older patients’ health and wellbeing, not only about disease. Currently the British Geriatrics Society Recommended Curriculum in Geriatric Medicine for Medical Undergraduates [19] and Oxford Medical School Geratology Curriculum do not include some of the specific topics listed by the patients (e.g. cancer, heart and respiratory conditions, isolation and loneliness).

### Strengths and limitations

A strength of our study is that it provides insight into areas where prior research is sparse. Another strength is the administration of the anonymised questionnaire on the day of their discharge, meaning patients were more likely to give honest answers.

One weakness of the study is not establishing what preparation for participation in teaching the patients received. Also, this was a single institution study with many questionnaires administered by medical students, introducing a possible bias that patients may not be willing to document their true thoughts with a medical student present. There was also no randomisation for which patients were asked to complete surveys, instead all eligible patients who gave consent and who were in the randomly chosen wards when the investigators were available to conduct the study were included.

Another weakness is that those patients who did not interact with students were not asked if this was because they refused to, or because they simply did not come across students during their admission. So while patients who did see students appeared more willing to see them again, it is unclear whether this is because the experience was a positive one, or because those unwilling to see students had already requested not to during that current admission.

## Conclusion

This study has several implications for educational practice in geriatric medicine; older inpatients reported positive experience with medical students education, although some outlined less favourable aspects, necessitating further elaboration and the development of a strategy to improve these. They were willing to participate in medical students education again, slightly less in invasive procedures, such as venepuncture. They believed that with the ageing population, it is more important than ever for students to learn from the older patients, and that older people should help educate the doctors of the future. Participating in medical student education increased older inpatients willingness for future involvement with students, suggesting that patients who feel that they had gained something from their interaction with the students are more receptive to further interactions. Educators should consider dedicating more time for student/older inpatient interactions and help students to improve their ability to listen, to give better and louder explanation and be more assertive. Geriatric Curriculum development should consider the inclusion of new topics like isolation and loneliness.
